# Novel Variant of Beilong Paramyxovirus in Rats, China

**DOI:** 10.3201/eid1806.111901

**Published:** 2012-06

**Authors:** Patrick C.Y. Woo, Susanna K.P. Lau, Beatrice H.L. Wong, Ying Wu, Carol S.F. Lam, Kwok-Yung Yuen

**Affiliations:** The University of Hong Kong, Hong Kong, People’s Republic of China

**Keywords:** viruses, paramyxoviruses, Beilong virus, molecular epidemiology, rodents, rats, China

**To the Editor:** In 2003, two cDNA strands were identified in a human mesangial cell line during experimental screening for genes upregulated by angiotensin II (*1*). Sequence analysis showed that the strands were homologous to the matrix, fusion, and phosphoprotein genes of paramyxoviruses, suggesting the possibility of a novel paramyxovirus (*2**,**3*). Subsequent research found that these sequences, believed to originate from human kidney mesangial cell lines, were not amplifiable from such cell lines or human kidney samples but were amplifiable from a rat kidney mesangial cell line (*4*). Isolation and complete genome sequencing of the virus confirmed that it was a novel paramyxovirus of the subfamily *Paramyxovirinae*, named Beilong virus (BeV).

BeV is most closely related to J virus, discovered in autoculture of kidney tissue from a moribund house mouse, and Tailam virus from Sikkim rats (*5**,**6*). Because J virus and Tailam virus were found to originate in rodents and BeV was amplifiable from a rat kidney mesangial cell line, we hypothesized that BeV was a novel paramyxovirus originating in rats. To test this hypothesis, we conducted a territorywide molecular epidemiologic study of rats and other mammals to evaluate this novel paramyxovirus.

We tested 4,130 samples from 1,398 animals collected from various locations in Hong Kong, People’s Republic of China, during September 2008–August 2009 (Table). These included 480 kidney, spleen, respiratory swab, and anal swab samples from 120 asymptomatic rats (105 brown rats [*Rattus norvegicus*] and 15 black rats [*R. rattus*]). To prevent cross contamination, we used disposable scalpels, decontaminated the work surface, and used sterile gloves for each tissue sample. We performed RNA extraction and reverse transcription PCR by using strategies we previously published for discovery and epidemiologic study of paramyxoviruses (*6**–**9*).

**Table:** Mammals screened for Beilong virus, People’s Republic of China, September 2008–August 2009

Animal	Sample type*
Bats, n = 502	Throat swab, rectal swab
Cats, n = 130	Nasal swab, rectal swab, urine, blood
Cattle, n = 100	Nasal swab, rectal swab, liver, buffy coat, plasma
Dogs, n = 149	Nasal swab, rectal swab, urine, blood
Hamsters, n = 49	Throat swab, intestinal swab, kidney
Pigs, n = 100	Nasal swab, rectal swab, liver, blood
Wild urban rodents, n = 120	Rectal swab, throat swab, rectal swab, kidney, spleen
Wild rural rodents, n = 248	Throat swab, rectal swab

*All sample types listed for each animal were collected except wild rodents: 237 throat swab and 246 rectal swab samples were collected from 248 wild rodents.

We performed BeV screening by PCR amplification of a 440-bp fragment of the large (L) gene, located at the 5′ end of the genome and used specific primers (LPW9739 5′-GGAGGATTCCCTCATAGAGAA-3′ and LPW9741 5′-CTCATATGTATTTACATTTAAACCA-3′). The PCR mixture (25 μL) contained cDNA, PCR buffer (10 mmol/L Tris-HCl, pH 8.3, 50 mmol/L KCl, 3 mmol/L MgCl_2_, and 0.01% gelatin), 200 μM of each dNTP, and 1.0 units of *Taq* polymerase (Applied Biosystems, Foster City, CA, USA). The mixtures were amplified in 60 cycles at 94°C for 1 min, 48°C for 1 min, and 72°C for 1 min; and a final extension at 72°C for 10 min in an automated thermal cycler (Applied Biosystems).

BeV in the positive samples was confirmed by amplifying a 318-bp fragment of the nucleocapsid (N) gene of BeV, located at the 5′ end of the genome; by using specific primers (LPW10723 5′-TATATGGTTGAGATYCTNATHGA-3′ and LPW10408 5′-CCATKGCRTAGCTCCADAG-3′) and experimental conditions described above. We confirmed the specificities of the primers by testing samples positive for Tailam virus (*6*), which all showed negative results.

Results of reverse transcription PCR for a 440-bp fragment in the large gene of BeV were positive for 40 kidney and 9 spleen samples from 43 rats (40 brown rats and 3 black rats). Sequencing and phylogenetic analysis showed 6–13 base differences between the sequences and the corresponding region in the large gene of BeV (GenBank accession no. NC_007803), suggesting that this is a novel variant of BeV in our locality.

Results of reverse transcription PCR for a 318-bp fragment in the N gene of BeV were positive in the same 40 kidney and 9 spleen samples from the 43 rats. Sequencing and phylogenetic analysis showed 1–9 base differences between the sequences and the corresponding region in the N gene of BeV. The kidney and spleen samples were positive in 4 brown rats and 2 black rats. The L and N gene sequences amplified from the kidney and spleen samples were identical in 5 of the 6 rats. However, in 1 brown rat, L and N gene sequences from the kidney and spleen samples differed by 4 and 6 bases, respectively, suggesting the possibility of 2 strains of BeV in the same rat. None of the samples from the other mammals were positive. The authenticity of the results was supported by identical results from 2 independent genes of the BeV genome, sequence variations in the L and N genes from the positive samples, and negative results from all other mammals tested.

This study suggests that BeV and its variants are endemic in brown rats and black rats, but it is not known whether transmission is vertical or horizontal. Detection of BeV and Tailam virus in kidney and spleen samples, but not respiratory or anal swabs, suggested that they are probably systemic viruses excreted in urine. Phylogenetic and genomic evidence support the grouping of BeV, Tailam virus, and J virus into a new genus of *Paramyxovirinae*. Distinctly, the genomes of all 3 viruses contain 8 genes (3′-N-P/V/C-M-F-SH-TM-G-L-5′). We speculate that the ancestor of these closely related paramyxoviruses infected the common ancestor of rats and mice, with subsequent co-evolution and divergence with the host.

## References

[1] Liang X, Zhang H, Zhou A, Wang H. AngRem104, an angiotensin II–induced novel upregulated gene in human mesangial cells, is potentially involved in the regulation of fibronectin expression. J Am Soc Nephrol. 2003;14:1443–51. 10.1097/01.ASN.0000067860.64692.C0 12761244

[2] Basler CF, Garcia-Sastre A, Palese P. A novel paramyxovirus? Emerg Infect Dis. 2005;11:108–12. 10.3201/eid1101.040653 15705331 PMC3294354

[3] Schomacker H, Collins PL, Schmidt AC. In silico identification of a putative new paramyxovirus related to the *Henipavirus* genus. Virology. 2004;330:178–85. 10.1016/j.virol.2004.09.019 15527844

[4] Li Z, Yu M, Zhang H, Magoffin DE, Jack PJ, Hyatt A, Beilong virus, a novel paramyxovirus with the largest genome of non-segmented negative-stranded RNA viruses. Virology. 2006;346:219–28. 10.1016/j.virol.2005.10.039 16325221

[5] Jack PJ, Boyle DB, Eaton BT, Wang LF. The complete genome sequence of J virus reveals a unique genome structure in the family *Paramyxoviridae.* J Virol. 2005;79:10690–700. 10.1128/JVI.79.16.10690-10700.2005 16051861 PMC1182632

[6] Woo PC, Lau SK, Wong BH, Wong YP, Poon RW, Yuen KY. Complete genome sequence of a novel paramyxovirus, Tailam virus, discovered in Sikkim rats. J Virol. 2011;85:13473–4. 10.1128/JVI.06356-11 22106385 PMC3233173

[7] Lau SK, Woo PC, Wong BH, Wong AY, Tsoi HW, Wang M, Identification and complete genome analysis of three novel paramyxoviruses, Tuhoko virus 1, 2 and 3, in fruit bats from China. Virology. 2010;404:106–16. 10.1016/j.virol.2010.03.049 20537670 PMC7111929

[8] Lau SK, To WK, Tse PW, Chan AK, Woo PC, Tsoi HW, Human parainfluenza virus 4 outbreak and the role of diagnostic tests. J Clin Microbiol. 2005;43:4515–21. 10.1128/JCM.43.9.4515-4521.2005 16145100 PMC1234116

[9] Lau SK, Li KS, Chau KY, So LY, Lee RA, Lau YL, Clinical and molecular epidemiology of human parainfluenza virus 4 infections in Hong Kong: subtype 4B as common as subtype 4A. J Clin Microbiol. 2009;47:1549–52. 10.1128/JCM.00047-09 19261793 PMC2681862

